# Neighborhood Walking and Social Connectedness

**DOI:** 10.3389/fspor.2022.825224

**Published:** 2022-04-12

**Authors:** Troy D. Glover, Joe Todd, Luke Moyer

**Affiliations:** The Department of Recreation and Leisure Studies, University of Waterloo, Waterloo, ON, Canada

**Keywords:** community, tie strength, civil inattention, strangers, neighborhoods and health

## Abstract

Neighborhood social ties matter crucially, especially during stressful life events like a global pandemic, for they represent vital sources of wellbeing and community capacity. Activities that enable community members to engage in incidental sociability and acts of “neighboring”—that is, authentic social interactions with their neighbors—warrant attention from sport and active living researchers because of their potential to bolster the social fabric of our neighborhoods and facilitate neighbors' access to important resources, such as information, material resources, and social support. Though perhaps dismissed as trivial, neighborhood walking represents a valuable and underappreciated everyday activity that fits this description, especially in an age characterized by an epidemic of social isolation and loneliness. Despite its vast potential to address the quasi-anonymity of urban life, neighborhood walking remains surprisingly underexamined as a facilitator for fostering social connectedness, the sense of connection and social bond people feel toward others. The goal of this manuscript, therefore, is to establish the conceptual grounding for how neighborhood walking strengthens social ties among neighbors to facilitate access to important coping resources. In doing so, it aims to advance a research agenda on walking that moves beyond the benefits of physical activity.

## Introduction

As a driver for its 2018 #coffeewithneighbours campaign, Tim Hortons, a ubiquitous fast food chain in Canada, reported that as many as half of Canadians do not know their neighbors (Tim Hortons, 2018). Other Canadian reports confirm these findings to reveal familiarity with neighbors extends to only a surprising few (Environics Institute, 2018). American data tell a similar story; the number of Americans with no confidants evidently tripled over a 30-year period (McPherson et al., 2006). These social trends, among others, put people at greater risk for health problems by constraining engagement in meaningful social interactions with others. Ultimately, the disconcerting state of neighborliness has serious consequences for people's health, for social ties serve as vital sources of wellbeing and community capacity to help people get by and get ahead in life, particularly during stressful life events (Glover and Parry, 2008).

Far from withdrawing from one another during the COVID-19 pandemic, many people, including vulnerable populations (i.e., older adults and newcomers), attempted to address their social isolation by participating in an ostensible resurgence in “neighboring”—that is, engagement (from a safe distance) in authentic social interactions with their neighbors, the people closest and most accessible to them geographically (see Glover, 2020). As a New York City resident commented in a New York Times article at the beginning of the pandemic (How the stoop the sidewalk helped New Yorkers stay sane, 2014), “The word ‘neighbor’ has taken on a new meaning. We now greet each other with more than a polite hello; we've crossed a line with each other.” This so-called “thickening of thin ties”—a strengthening of our social connections—warrants attention because it gives neighbors important access to necessary social resources to cope with their circumstances.

While research on the physical and mental health benefits associated with walking pervade in the active living literature (Lee and Buchner, 2008), the social benefits of walking have garnered less attention (Leyden, 2003; McCain, 2021). Researchers do link walkable neighborhoods (i.e., the physical features of a neighborhood that make it walkable) to the generation of social capital (Leyden, 2003) and sense of community (Wood et al., 2010), but few studies focus on the actual act of walking and its connection to social connectedness. Even where research examines walking as a social practice—that is, as “a socially organized, embodied activity ‘bundled’ with material arrangements and linked into a nexus by understandings, rules, and teleo-affective structures” (Harries and Rettie 2016, p. 875; see also Schatzki, 2010)—empirical links to social connectedness remain surprisingly underexplored, if explored at all. Despite its vast potential to address isolation in urban life, neighborhood walking remains surprisingly underexamined as a facilitator for social connectedness, what Haslam et al. (2015, p. 1) defined as “the sense of belonging and subjective psychological bond that people feel in relation to individuals and groups of others.”

While the COVID-19 pandemic undoubtedly posed a serious challenge to personal networks of close relations, it had an even greater impact on our social connectedness by limiting our incidental sociability with people we recognize, but do not know. With our daily routines altered, the pandemic changed our mobility patterns, keeping us from encountering others whose routines overlapped with our own. With many having localized their movements to places closest to home, neighborhood walking emerged as one of the key activities that facilitated neighboring and enabled the strengthening of neighborhood social ties (Glover, 2020; Mehta, 2020). In doing so, it established itself as a promising area of research that warrants attention from our field.

Accordingly, the purpose of this paper is to establish the conceptual grounding for how neighborhood walking strengthens social ties among neighbors to facilitate access to important coping resources. In this sense, it aims to advance a research agenda on walking that moves beyond its current focus on physical activity and mental health. With this agenda in mind, we begin this paper by establishing social isolation and loneliness (i.e., the lack of social connectedness in society) as a serious social problem in need of attention. We then discuss public space as an important realm in which we routinely encounter other people, and identify neighborhoods, in particular, as meaningful spaces of social connection. Finally, we explore walking as a tactic to facilitate neighborhood connections, which leads to our call for a research agenda focused on neighborhood walking and social connectedness.

## The Scourge of Social Isolation and Loneliness

Social isolation and loneliness persist alarmingly across the globe as increasingly serious and complex health problems. Social isolation refers to “the actual absence of informal supportive relationships,” whereas loneliness describes “a subjective and negatively experienced discrepancy between the quality and quantity of existing relationships and a person's desires or standards with regard to relationships” (Machielse, 2015, p. 340). Loneliness, in other words, can be thought of as *subjective* social isolation. Troublingly, Holt-Lunstad (2020, p. 2) reported, “Even by the most conservative estimates, loneliness affects one in five adults.” Consequently, its pervasiveness should be cause for grave concern, given that robust evidence from multiple meta-analyses and large-scale prospective epidemiological studies shows a lack of social connection significantly increases risk for morbidity and early mortality (Holt-Lunstad et al., 2010). All told, social connectedness affects our health and wellbeing significantly, and its absence can result in harmful outcomes for those who feel a lack of connection to others (Pinker, 2014).

As if a lack of social connectedness were not already a major societal problem, the arrival of COVID-19 in 2020 led to workplace closures and so-called “social distancing” measures that limited physical contact among people to mitigate the spread of infection, thereby threatening to intensify already high levels of social isolation and loneliness. Physical restrictions implemented to “flatten the curve,” while intended paternalistically to limit exposure to the virus, ultimately constrained mobility, restricted people to isolate at home (sometimes by themselves), and kept people from seeing family and friends in person. While encouraged to “socially distance,” paradoxically, people needed more than ever to connect socially with others.

Evidence of the continued persistence of loneliness during the pandemic reveals its stubborn durability as a social problem. Heidinger and Richter (2020) found loneliness in Australian adults aged 60 and older increased slightly from 2019 to 2020. Similarly, van Tilburg et al. (2021) observed an increase in loneliness among Dutch adults aged 65 and older during the pandemic, and Krendl and Perry (2021) reported a slight increase in American older adults' loneliness from 2019 to 2020. Interestingly, a Canadian study found levels of social isolation failed to change during the pandemic (Folk et al., 2020), possibly because digital contact with others helped many Canadians compensate for their lack of face-to-face contact (Peng and Roth, 2021). Even so, certain groups of people proved to be more vulnerable than others to changes in social isolation and loneliness during the pandemic, including youth, older adults, women, and those with chronic conditions (Luchetti et al., 2020). Whatever its social impact, however, COVID-19 seemingly made people recognize the importance of their relationships, for they appeared to prioritize their social connections and became more aware and appreciative of the relationships in their lives.

Even with an ostensibly greater recognition of the importance of social connection that emerged from the pandemic, social isolation and loneliness continue to persist disconcertingly and globally as widespread health problems. Finding ways to help those battling these serious health matters warrants attention, especially in the age of COVID-19, but in its eventual aftermath, too. Neighborhood walking offers one potential solution. While we know the act of walking has social benefits (McCain, 2021), research on its direct relationship with social connectedness is surprisingly sparse. Accordingly, this paper explores its potential and offers the field of active living direction in moving forward in combatting the scourge of social isolation and loneliness.

## Neighborhoods as Spaces of Connection

Under normal circumstances, most people engage in an endless number of encounters with others in their daily experiences of modern urban life. These interactions, for the most part, occur anonymously (Hirschauer, 2005) and with a sense of detachment. To be fair, we cannot know everyone, so most social interactions among urban inhabitants in the public realm resemble something Goffman (1963) referred to as *civil inattention*, a normalized display of disinterestedness without contempt. By being inconspicuous, strangers who encounter others make their intentions to avoid social interaction clear, thereby transforming public spaces into quasi-private ones (Cooper, 2007). Remaining unknown to others sidesteps any meaningful interaction to deter the possibility of “acquaintanceship” and inhibit any obligation produced by the encounter (Goffman, 1963). Not surprisingly, then, treating each other like strangers in public normalizes strangeness (Hirschauer, 2005) and gives people the feeling they are isolated or lonely.

Routine encounters over time, however, can give rise to relations of “familiar strangers” (Ahmed, 2000). While a stranger refers to “someone who has not been knowingly encountered before” (Cooper 2007, p. 205), a *familiar stranger* denotes “an individual who is recognized from regular activities, but with whom one does not interact or communicate” (Jackson et al. 2017, p. 9). Also referred to as “invisible ties” or “nodding relationships,” these anonymous, albeit recognizable social connections “become known over time and are no longer interchangeable” (Felder 2020, pp. 7–8). Amazingly, with repeated exposure, these fleeting and routinized relationships can transform even further into more sociable encounters that contain varying degrees of warmth, rapport, and intimacy (Lofland, 2017). Relationships among social ties, in other words, are not static, but rather fluid insofar as they often exist within a process of transformation (Lofland, 2017). Exploring this transformation (i.e., the strengthening/weakening of social ties) offers active living scholars a lens for understanding how activities such as walking contribute to social connectedness and its dynamic nature.

Neighborhoods, with their streets, sidewalks, and open spaces, represent meaningful settings within the public realm that facilitate routine encounters in which social ties can strengthen (or wither). Relationships within neighborhoods can span the spectrum of social ties from strong to weak to invisible (Felder, 2020). Put another way, neighbors can be close friends, acquaintances, or nominal individuals whom we fail to see at all (Rosenblum, 2016; Felder, 2020). They can also be negative or hostile. Whatever the nature of our ties, the neighbors we identify in our neighborhoods register with us, not just because of their physical proximity to us, but also because we have *knowledge* of them. Consequently, some fit into the imagined geography of our neighborhood (Rosenblum, 2016), while others do not.

So-called walkable neighborhoods seemingly provide material form that increases the likelihood of social interactions between and among inhabitants (Kim and Kaplan, 2004; Glanz, 2011). Talen and Koschinsky (2013, p. 43) defined a walkable neighborhood as “a safe, well-serviced neighborhood, imbued with qualities that make walking a positive experience,” qualities such as urban form that encourages pedestrian activity and minimizes environmental degradation; social, economic, and land use diversity; connected uses and functions; a quality public realm that provides opportunities for interaction and exchange; equitable access to goods, services, and facilities; and protections that advance environmental and human health (p. 44). Complete or traditional neighborhoods (see New Urbanism) in which pedestrians do not compete with cars, moreover, encourage walking (Appleyard, 1970; Leyden, 2003). Ultimately, advocates of walkable neighborhoods highlight the difference incidental sociability facilitated by the material form of a neighborhood makes for meaningful social interaction. As Leyden (2003, p. 1546) wrote:

Spontaneous ‘bumping into’ neighbors, brief (seemingly trivial) conversations, or just waving hello can help to encourage a sense of trust and a sense of connection between people and the places they live. These casual contacts can occur at neighborhood corner shops, at local parks, or on the sidewalk. To many residents, such contacts breed a sense of familiarity and predictability that most people find comforting.

In less romantic terms, Sennett (1971) referred to this form of interaction as *social friction*, the little inefficiencies that force people to interact with strangers. Admittedly, the greater the perception of strangers within a neighborhood, the less inclined individuals are to interact with them socially (French et al., 2014). Along these lines, Putnam (2007) revealed how neighbors, when faced with the perception of overwhelming diversity, often “hunker down” to avoid contact with others. Whatever the response to strangers, active living researchers, by focusing on neighborhood walking and social connectedness, have an opportunity to explore whether intergroup contact while walking their neighborhoods lessens prejudice (contact hypothesis), increases it (conflict hypothesis) or avoids contact altogether (constrict hypothesis). No matter the finding, facilitating social interaction—the “formal (e.g., active, planned) or informal (e.g., casual, unplanned) social opportunities during which two or more people attend to the quality of their relationships” (Kim and Kaplan 2004, p. 316)—presumably matters as a first step toward building a greater sense of community among urban inhabitants.

In this manuscript, we argue that neighborhood walking plays a particularly meaningful role in facilitating social connectedness, and in doing so, we aim to advance a new area of research that has received surprisingly little attention in the literature. While an impressive number of studies focus on perceptions and features of the walkability (i.e., design) of a neighborhood and their association with social capital development (see Leyden, 2003; Hanibuchi et al., 2012), the actual impact of the social experience of walking the neighborhood has received scarce attention. Interestingly, Lund (2003) found no significant direct relationship between objective environmental variables and acts of neighboring. Moreover, after examining the proposition that more walkable neighborhoods encourage local social interaction, Du Toit et al. (2007) concluded influences on neighborhood sociability extend beyond issues of urban form. While attention to the built environment (i.e., walkability) remains an important area of research, a focus on neighborhood social processes, such as engaging in neighboring through the activity of neighborhood walking deserves much greater consideration from active living scholars.

## Walking as a Facilitator of Social Connections

The benefits of walking are well-documented. A strong body of literature demonstrates its positive impacts on physical health. If engaged in at recommended levels (i.e., for a minimum duration of 30 min at a minimum frequency of 5 days/week and at a moderate intensity), walking can play a role in managing coronary heart disease, hypertension, type 2 diabetes, obesity, elevated cholesterol, osteoporosis, and osteoarthritis (see Lee and Buchner, 2008). In addition, it has been posed seriously as a potential means to prevent dementia [see Federal Interagency Forum on Aging-Related Statistics (US)., 2004]. Furthermore, walking is relevant to addressing obesity by producing increases in caloric expenditure (Lee and Buchner, 2008).

More recently, scholars have begun lauding its positive contributions to mental health. Many point to its restorative and therapeutic properties (Doughty, 2013). Green (2009, p. 27) described it as providing “a route to reclaiming a particular consciousness, which involves some of the gains of a slower, more meditative way of life.” Indeed, researchers highlight its ability to encourage introspective reflection (Wylie, 2005; Edensor, 2010). It also enables escape from the impact of everyday living, including work-related and household responsibilities (Roberson and Babic, 2009). In this sense, it can introduce a sense of perspective, releasing the mind and “open[ing] up the senses to allow the re-calling of incidents, feelings and experiences that were constitutive of that individual's understanding of the life world” (Anderson (2004, p. 258). Ultimately, Solnit, 2001) argued walking aligns the mind, body, and world.

Unquestionably, walking represents a popular activity (Ekkekakis et al., 2008). It tends to be accessible insofar as it can be done almost anywhere, and it does not require expensive or specialized equipment, dedicated facilities, or extraordinary skills of any kind. It can also be done indoors or outdoors, making it incredibly versatile as an activity. Given its versatility, Lee and Buchner (2008, p. S516) wrote, “walking is particularly important for its potential to reduce disparities in health related to lack of physical activity.” They also noted its standing as activity with a low risk of injury (Lee and Buchner, 2008), making it particularly attractive for people across the lifespan.

Germane to the agenda we wish to advance, walking a neighborhood route, though perhaps undertaken for physical activity, to clear one's mind, or for fresh air, brings about increasing encounters with neighbors (i.e., micro-exchanges), including with those who are either previously unknown or recognized from regular activities, but with whom one does not typically interact or communicate (Rogers et al., 2011). As a slow-moving activity that lets walkers “take in” their neighborhood as they walk through it, neighbors attend to what is going on in their surroundings, thereby creating opportunities for social interaction and the strengthening of neighborhood ties. At worst, when neighbors repeatedly subject themselves to and observe each other while walking their neighborhood, the minimal social contact involved (e.g., nodding or a simple “hello”) increases their public familiarity (Rietveld et al., 2019). The shared daily path of a neighborhood walk makes neighbors more recognizable. Moreover, neighbors who see each other routinely become more visible, potentially transforming into familiar strangers or possibly even acquaintances or eventually friends, as time goes by. Connections thicken as interactions become more regular and frequent. What begins as “routinized relations” established during casual walks can turn into something more meaningful (Lofland, 2017).

Lund (2002) found a strong association between the frequency of walking within neighborhoods and unplanned interactions with neighbors, which contributes to relationship formation and development. For Giles-Corti et al. (2005), walking involves three dimensions: (1) a utilitarian dimension (i.e., as a necessary activity); (2) a leisure dimension (i.e., as an optional and recreational activity), and (3) a social dimension (i.e., as a vector of contact and interaction between individuals). Interestingly, Wood et al. (2010) found sense of community was related to walking, but only leisure walking, not brisk walking. In Lund (2002, p. 310) words:

Whereas strollers may be choosing to walk through their neighborhood because they feel like being apart of the neighborhood or they feel like running into and maybe even socializing with their neighbors, destination walkers may more often be walking purely out of necessity or under time constraints. They may not feel like being, or have the time to be, “disturbed” by their neighbors or to enjoy their surroundings. They are also more likely to be limited in their route choices. Whereas strollers can choose the safest and most pleasant route, or the one where they know they are more likely to run into a neighbor, destination walkers will typically choose the most direct route. If this route is not as pleasant as they may wish, this may contribute to a decreased sense of community.

In this sense, understood as a social practice, neighborhood walking can take the form of a dispersed practice or an integrative practice (see Harries and Rettie, 2016). The former “center[s] around a single type of action” (Schatzki 2010, p. 88): walking as an end-in-itself or walking as a practice in which walking is what is being achieved (e.g., destination walkers). The latter, by contrast, carries teloaffective elements—what Schatzki (2010, p. 89) described as “ends, projects, tasks, purposes, beliefs, emotions, and moods.” These set the rules for how walking ought to be performed under certain circumstances so that neighborhood walking becomes a means-to-an-end (e.g., strollers)—that is, walking within practices in which walking itself is secondary to its ultimate goal (e.g., socializing).

But even if walking only leads to greater, albeit restrained, familiarity with others, that familiarity forms the core of well-functioning neighborhoods (Rietveld et al., 2019). Greater familiarity generates what Horgan (2012, p. 619) referred to as “soft solidarity,” a form of mutuality recognized and sustained by apparent strangers without a requirement of explicit recognition. It also, at a minimum, engenders the surprisingly health sustaining feeling of *mere belonging*, a minimal social connection to another person or group (Walton et al., 2012). Irrespective of the outcome, neighborhood walking expands our imagined geographies, making our neighborhoods better places to live. When neighbors stop to talk with each other from across the street, they invite meaningful social interaction and create an opening to build a relationship, even if only superficially. They no longer see themselves as strangers participating in random encounters. By noticing, acknowledging, and engaging with neighbors (i.e., neighboring), “feelings of solidarity, increases in emotional energy, creation of symbols, and feelings of morality all stem from [the] interaction” (Campos-Castillo and Hitlin 2013, p. 170). Welcoming a neighborly interaction, even if only for a brief, albeit authentic, moment establishes a bond of mutual obligation, which opens the relationship up to future engagement and potential favors (Rosenblum, 2016).

The actions facilitated by this social capital make neighbor relations a vital part of creating community capacity. By social capital, we mean “the consequence of investment in and cultivation of social relationships allowing an individual access to resources that would otherwise be unavailable to him or her” (Glover et al. 2005, p. 87). First, social capital facilitates *expressive action* (or getting by), which refers to emotional support (Lin, 2001). Relationships within a neighborhood assist neighbors in coping with their life situations through the receipt of empathetic support, such as a hug or phone call to check in on someone who lives alone. This sort of support usually comes from our strong ties (Lin, 2001). Second, social capital enables *instrumental action* (or getting ahead), the material dimension of our neighborhood relations (Bourdieu, 1986; Lin, 2001). It gives neighbors access to resources, such as invitations to parties, opportunities to borrow, or referrals to connect with other social contacts, that help them advance their social position. Access to these resources tend to come from our weak ties because they fall outside of our immediate social circle and have access to different resources (Lin, 2001). And third, the darker side of social capital can make possible *obstructive action* (or falling behind), which refers to actions taken by neighbors that work against their own interests (Glover and Parry, 2008). In other words, neighbors can face peer pressures that result in unhealthy behaviors or actions (e.g., ostracizing other neighbors). To make all of these actions possible, neighbors activate the social capital that exists within their neighborhood networks. Engaging in greater civil attention in the public spaces of our neighborhoods (i.e., sidewalks, streets, greenspaces), therefore, has the potential to increase our capacity for resilience by improving our functioning and adaption during times of adversity (Norris et al., 2008) and social isolation (Glover, 2018). However, as Cattell et al. (2008, p. 546) noted “different kinds of ties [fostered in public space] are likely to carry different implications for both well-being and for community integration.” This research agenda endeavors to understand how neighborhood walking contributes to the development of different kinds of ties and the resources those ties make available to neighbors, a subject that remains surprisingly underexamined.

## Neighborhood Walking and Social Connectedness: A Research Agenda

We believe a robust research agenda on neighborhood walking and social connectedness will only add to the social relevance of active living research. To be sure, questions associated with the role(s) neighborhood walking plays in strengthening local social ties and building social capital warrant attention from active living scholars. In advancing this line of research, however, we recognize it may likely ignore people who are unable, for one reason or another, to go for routine walks in their neighborhoods. Accordingly, we suggest researchers who wish to contribute to this research agenda necessarily recognize, critically reflect upon, and trouble the three prerequisites Stevenson and Farrell (2018, p. 432) identified with taking a leisure walk: free time, a place to go, and a body unhindered by illness or social restraints. Each presents important considerations for those interested in examining neighborhood walking and social connectedness.

First, *free time* underscores the importance of acknowledging class and gender differences in having the privilege to make time available to engage in neighborhood walking. As Teixeira et al. (2014) reported, people of lower socio-economic status are less likely to walk for leisure and more likely to walk to commute than those with higher socio-economic status. During the pandemic, those able to work remotely from home could make time to go for a leisure walk, whereas others whose livelihoods required them to work in-person did not have the same privilege. This observation concurs with Davies et al. (2012) who identified work patterns as a key constraint to leisure walking, a finding that mirrors general trends observed in the active living literature inasmuch as working longer hours reduces the likelihood of engaging in regular exercise (Popham and Mitchell, 2006). Time of day also constrains certain people from walking in their neighborhoods. The threat of darkness and its implications for a sense of safety often discourages women, in particular, from using public spaces, including neighborhood sidewalks, at night (Yen et al., 2007). These time-related constraints warrant consideration in research on neighborhood walking and social connectedness.

Second, *having a place to go*, though seemingly obvious, assumes individuals have access to a walking environment in which they feel safe and welcome. Of course, not everyone has access to neighborhoods conducive to a pleasant, sociable walking experience. Indeed, while the COVID-19 pandemic made clear the broader social relevance and importance of the public realm, especially for marginalized populations that sought refuge in places that enabled them to enjoy a leisurely break or time away from their lockdown experience (Wray et al., 2020), not everyone enjoyed equal access. To be sure, inequalities in space during the pandemic made self-isolating and physical distancing challenging. Public spaces, if even available, were often the only outdoor leisure spaces marginalized groups could access and use to get relief from their crowded living conditions (Honey-Rosés et al., 2020). Even where such spaces were geographically accessible, those living in deprived areas were more likely to view them as unsafe (Honey-Rosés et al., 2020). “If people are fearful,” wrote Wood et al. (2008, p. 16), “they may be less likely to go out of their home, use local facilities, attend clubs or functions or interact with strangers or people they meet ‘in the street’, particularly at night.” More than a crisis of public order, then, the pandemic showed itself to be a crisis of environmental justice and equity (Wray et al., 2020). The pandemic also saw differences in enforcement of public health restrictions, whereby their implementation impacted Black, Indigenous, and people of color disproportionately. “The reality of our public green and open spaces,” Hoover and Lim (2021, p. 63) explained, “is that Black and brown people are more heavily policed and surveilled, leading to arrests, citations, and in some cases, death.” These issues of inequality warrant serious attention for researchers interested in exploring neighborhood walking and social connectedness.

Third, having a body unhindered by illness or social restraints reveals serious constraints to neighborhood walking by those with disabilities, including many older adults. Alidoust et al. (2018, p. 134) confirmed that older people tend to avoid walking when they believe their neighborhoods are restrictive to their mobility. They listed long distances, lack of resting places, noisy traffic, dangerous crossroads, steep terrain, and streets in poor condition as constraints to participation in walking by many older adults. Along these lines, those with disabilities unsurprisingly perceive fewer neighborhood environmental supports and more barriers for walking than their non-disabled counterparts (Omura et al., 2020). Furthermore, though no harmful effects are associated with walking for those with social anxiety (see Kelly et al., 2018), the potential for social encounters may dissuade such individuals from venturing out locally. Neighborhood walking, in short, is not possible for everyone, which deserves recognition by anyone considering examining its relationship with social connectedness.

In addition to recognizing the constraints identified above, future research should consider the implications of walking together with another person, other people (see Gilbert, 1990), or with dogs, in contrast to walking alone. Stevenson and Farrell (2018, p. 442) found the majority of their research participants viewed walking as a social experience associated with meeting friends, conversation, enjoyment, and shared contemplation. The rhythms associated with walking together with others, they surmised, provided opportunities for participants to alternate between conversation and silent thought and reflect together and alone. Similarly, walking dogs deserves attention for its role in connecting people during walking, given the propensity for dog walkers to walk more than non-dog walkers (Brown and Rhodes, 2006).

While results remain mixed about whether neighborhoods deemed more walkable afford and encourage more social interaction (Leyden, 2003), the actual relationship between the act of walking and sociability needs attention. We believe the agenda outlined in this paper can build toward an important body of work that further positions active living as a relevant area of social science inquiry. The unfortunate health consequences of social isolation and loneliness, as observed before and during the pandemic, should inspire active living researchers to study physical activities that draw people together and encourage greater human connection.

## Data Availability Statement

The original contributions presented in the study are included in the article/supplementary materials, further inquiries can be directed to the corresponding author.

## Author Contributions

TG drafted the manuscript and received supportive contributions from the co-authors. All authors contributed to the article and approved the submitted version.

## Funding

This article stems from a research project titled Neighboring on the Move: Walking the Neighborhood, Strengthening Social Ties, and Building Social Capital funded by an Insight Grant from the Social Sciences and Humanities Research Council of Canada, file number 435-2021-0571.

## Conflict of Interest

The authors declare that the research was conducted in the absence of any commercial or financial relationships that could be construed as a potential conflict of interest.

## Publisher's Note

All claims expressed in this article are solely those of the authors and do not necessarily represent those of their affiliated organizations, or those of the publisher, the editors and the reviewers. Any product that may be evaluated in this article, or claim that may be made by its manufacturer, is not guaranteed or endorsed by the publisher.
